# A qualitative study of primary care clinicians' views of treating childhood obesity

**DOI:** 10.1186/1471-2296-8-50

**Published:** 2007-09-03

**Authors:** Olivia Walker, Mark Strong, Rebecca Atchinson, Joanna Saunders, Jo Abbott

**Affiliations:** 1 University of Sheffield Medical School, Beech Hill Road, Sheffield, S10 2RX, UK; 2 School of Health and Related Research, University of Sheffield, Regent Court, 30 Regent Street, Sheffield, S1 4DA, UK; 3 Rotherham Primary Care Trust, Oak House, Moorhead Way, Bramley, Rotherham, S66 1YY, UK

## Abstract

**Background:**

The prevalence of childhood obesity is rising and the UK Government have stated a commitment to addressing obesity in general. One method has been to include indicators relating to obesity within the GP pay-for-performance Quality and Outcomes Framework (QOF) contract. This study aimed to explore general practitioners' and practice nurses' views in relation to their role in treating childhood obesity.

**Methods:**

We interviewed eighteen practitioners (twelve GPs and six nurses) who worked in general practices contracting with Rotherham Primary Care Trust. Interviews were face to face and semi structured. The transcribed data were analysed using framework analysis.

**Results:**

GPs and practice nurses felt that their role was to raise the issue of a child's weight, but that ultimately obesity was a social and family problem. Time constraint, lack of training and lack of resources were identified as important barriers to addressing childhood obesity. There was concern that the clinician-patient relationship could be adversely affected by discussing what was often seen as a sensitive topic. GPs and practice nurses felt ill-equipped to tackle childhood obesity given the lack of evidence for effective interventions, and were sceptical that providing diet and exercise advice would have any impact upon a child's weight.

**Conclusion:**

GPs and practice nurses felt that their role in obesity management was centred upon raising the issue of a child's weight, and providing basic diet and exercise advice. Clinicians may find it difficult to make a significant impact on childhood obesity while the evidence base for effective management remains poor. Until the lack of effective interventions is addressed, implementing additional targets (for example through the QOF) may not be effective.

## Background

### Context

Obesity is a complex public health issue representing a major threat to children's health [1]. Forecast projections suggest that by 2010 the proportion of children aged two to 15 who are obese will have risen to 19% in boys, and to 22% in girls [2]. The UK Government has responded by setting targets that aim to "halt the year on year rise in obesity among children aged under 11 by 2010 in the context of a broader strategy to tackle obesity in the population as a whole" [3]. As part of this broader strategy obesity was included in the general practice Quality and Outcomes Framework (QOF) contract for 2006–7 [4]. Under the QOF contract practices are rewarded if they can produce a register of patients aged 16 years and over who are obese. However, the number of QOF points assigned to this indicator is less than 1% of the total, perhaps undermining obesity's importance. Clearly there is the potential for other obesity related indicators to be included in future QOF contracts, and it is possible that these may relate to health outcomes, rather than processes. This is difficult territory: linking GP practice income to health outcomes that depend on the choices that patients make is controversial [5]. GPs may also resist further targets related to childhood obesity given that the evidence base in this area is so poor. A Cochrane systematic review of interventions for treating childhood obesity included 18 studies of various different treatments, but found little firm evidence of effectiveness for any of them [6]. Not surprisingly guidance for the prevention and treatment of obesity published by the UK's National Institute for Health and Clinical Excellence (NICE) strongly states the urgent need to develop this evidence base [7].

### Clinicians views of managing adult obesity

General practitioners' (GPs') views concerning their role in the management of adult obesity have been explored in a number of studies from a range of countries. One UK study concluded that general practitioners believed that obesity was not within their professional domain, even though patients wanted their doctor to take responsibility for their weight problems [8]. Other studies however, including another from the UK [9], have reported that GPs do feel they have a role in the management of obesity, either as counselling patients on health risks [9,10], or giving advice on weight management [11,12]. Regardless of whether GPs feel they have a role in the management of obesity, they are generally pessimistic about the likely impact of any advice that they give. A lack of evidence based interventions, a lack of training (particularly nutrition training), poor motivation on the part of the patient and poor family support have been cited as important reasons for failure [8,11,13]. It is not surprising then that many GPs find managing obesity unrewarding or frustrating [9,12]. In contrast, research with practice nurses has found that they generally felt confident in giving weight loss and nutritional advice. However, they were not optimistic that patients would follow this advice, or that weight loss would result [14].

### Clinicians views of managing childhood obesity

A number of studies of clinicians' attitudes relating to childhood obesity have been conducted [15-18]. Managing childhood obesity has the potential to be more complex than managing adult obesity because the clinician is interacting not only with the child, but the wider family as well. Parents may not recognise or accept that their child has a weight problem [19,20], and GPs feel that even by raising the issue a breakdown in the doctor patient relationship may result [18]. A child's weight is potentially a very sensitive subject given the link between nurturing and feeding, and the relationship between body size and self image [18]. Despite these difficulties Australian GPs in one study felt optimistic that they could make a difference [15], perhaps suggesting important differences in attitude towards obesity between countries. In this study we aimed to explore the views of GPs and practice nurses concerning childhood obesity in one district in the north of the UK.

## Methods

### Participants

In May 2006 the practice managers from the 39 general practices who contract with Rotherham Primary Care Trust were asked to invite their GPs and practice nurses to participate in this study. Eighteen participants from 11 practices responded to the invitation, of which 12 were GPs (11 male and 1 female) and six (all female) were practice nurses. The majority of the participants were aged 40–49 years (30–39 years, n = 4; 40–49 years, n = 10; 50–59 years, n = 4). The twelve practices from which the participant GPs and nurses were drawn varied in terms of their size, and the socioeconomic status of the registered patient population. Socioeconomic status, as measured by the practice population weighted mean Index of Multiple Deprivation 2004 score [21], ranged from 17.7 to 37.6 with a mean of 29.2, and the practice list sizes varied from 4,589 to 17,669 with a mean of 9,554.

### Data collection and analysis

Data collection was by semi-structured interview following the interview schedule used by Epstein and Ogden, adapted to relate to childhood rather than adult obesity [8]:

Think about the last time you had a consultation with a parent/child who expressed concerns over their child's weight...

OR

Think about the last time you were in a consultation with a child and you expressed concerns about their weight...

Can you tell me about the consultation?

How did you feel about managing this patient?

What advice or information did you provide for the patient/their parent?

What did you think the patient/their guardian expected from you?

Did you feel that the consultation was successful?

Do you think primary care has a role in dealing with childhood obesity?

What management do you think primary care should employ in tackling obesity in children?

How would/do you feel about managing children with obesity routinely?

As a GP/practice nurse, whom would you contact for support and advice in relation to obesity?

How do you feel about the following:

• Counselling in primary care?

• Behavioural adjustment techniques?

• Education in obesity management for GPs and practice nurses?

• Extending the primary care team to include nutritionists and/or dieticians?

• Secondary and tertiary care in relation to obesity management?

Each interview, carried out face to face by researcher OW, lasted approximately 30 minutes. All interviews were recorded and transcribed verbatim. The transcribed data were analysed using the Framework method [22]. OW, after an initial stage of familiarisation with the data, listed key ideas and recurrent themes. The thematic framework was then refined in an iterative manner during subsequent readings of the transcripts. Data were annotated and indexed according to the emerging themes, and finally the data set was mapped and interpreted as a whole. A random sample of nine transcripts were similarly analysed by RA and the results were discussed between the two researchers in order to achieve a consensus view.

### Ethics

Our analysis of the views of primary care staff took place as part of a wider Rotherham PCT obesity service evaluation. As a service evaluation it did not require formal NHS ethical approval. This was confirmed by the chair of the South Yorkshire NHS Ethics Committee and the chair of the Rotherham Primary Care Trust Research Governance committee.

## Results

A number of themes emerged from the data. In summary, clinicians felt that families or other agencies, rather than the GP, were responsible for solving this difficult and often sensitive problem. Interventions were framed in terms of providing dietary and exercise advice, but these were felt to be ineffective. Lack of time was cited as a reason for sometimes avoiding engagement with the issue.

### The responsibility for managing childhood obesity

GPs and practice nurses felt that their role was confined to *raising *the issue of a child's weight with his or her parents, and managing any associated medical problems.

'Certainly our role is to raise it with parents... but not our role to see them every few months and ask, "how are you getting on". That is ultimately the parents' issue' (GP.1)

'I don't think we are managers of obesity, but really just to manage it in terms of co-morbidity or high risk factors' (GP.2)

The responsibility for *solving *the problem of obesity, however, was not felt to lie with the clinician. Responsibility lay either with the family, or with a public health agency:

'At the end of the day, parents have to be the ones to do it. They control what their child eats' (PN.3)

'It's a social problem and cultural problem and it's a family problem as well. It's got to be a big public health drive, a campaign to inform people really' (GP.5)

'I feel that there should be a different body other than the NHS that deals with well people, but that have these problems' (GP.4)

But it was also recognised that although the family may hold the key to solving the problem, they may be unwilling or unable to take on this responsibility:

'They tend not to accept they have a problem and it's very difficult to get them to stop eating junk food' (GP.5)

'They don't take any responsibility' (GP.11)

### The difficulty of the task

Childhood obesity was sometimes felt to be a problem that was just too difficult to tackle, and that clinicians could not cope with the scale of the problem. This may explain the unwillingness that clinicians felt towards accepting responsibility for it:

'We probably don't deal with obesity as often as we should, largely because I suppose we think it's difficult to deal with, there is no treatment as such' (GP.12)

'... I'm a bit numb to it because it seems so widespread' (GP.8)

'Perhaps we don't feel capable of coping with childhood obesity, we don't know what to do really' (GP.9)

GPs framed their interventions in terms of providing diet and exercise advice for the child and their family, but there was a feeling of pessimism that the advice would have little impact upon the child's weight:

'We try to reinforce the message of eating less and exercising more and giving advice on healthy diet. It's a pretty fruitless task really' (GP.3)

'We spend time with a family discussing diet and exercise... but they are not generally enthusiastic about diet and exercise regimes' (GP.10)

'Do they listen to you? No. I am very pessimistic about getting people to eat less' (GP.1)

It was felt that there was just too big a gulf between a healthy diet and what the children actually ate:

'Their perception of a good diet is so far adrift from what anyone else would consider a normal diet' (GP.5)

'The diet is difficult as there is often deep seated ignorance... about what are good foods and what are bad foods. The kids come in and they have got a packet of crisps in their hand or they are chewing on a mars bar' (GP.8)

'We talked about "five a day", this kid didn't eat five a week. Sunday lunch was the only time they ate vegetables. I have no great expectations that this kid will come back walking to school, eating "five a day", and lost any weight. I have very little faith' (GP.7)

### The sensitive nature of the subject

The problem is uncomfortable to deal with for several reasons. Firstly, GPs and nurses do not want to upset the child or the parent:

'I don't know how often I spontaneously bring it up, especially if the child is over five or six years of age and is in a position to understand me saying that they are overweight... I think, being honest, there is an element of not wanting to upset the child or the parent, despite the fact that I have no problem if an adult comes in, I'm quite blunt with them, but it seems harder with children' (GP.8)

'It's a hard thing to tackle and a hard thing to tell a mum that her child's obese' (PN.5)

Some reasons for the sensitivity of the subject were given. The link between feeding and nurturing was made by one GP:

'They seem to like fat babies still, think they are healthy. They don't seem to have any notion that it's reasonable to restrict a baby's food, keep their weight under control... they usually are quite prickly about it and the reason is they are usually all fat' (GP.5)

The anxiety that bringing attention to a child's weight could cause psychological problems was also voiced:

'I think you have to be careful in how you approach it [a child's weight] as you don't want it to become an issue with the child' (PN.3)

Clinicians were concerned that they may jeopardise their relationship with the family if they mentioned the problem of a child's weight:

'Should we? [bring up the issue of a child's weight] Probably yes, but we don't. Usually because the response back is very negative. You don't want to lose their trust in the condition they have come to see you about. If you start talking about something else, you are seen to be criticising them, you lose the consultation, they go see someone else, and you lose the continuity of care' (GP.3)

Losing the trust of the family would be very damaging since the family is key to solving the problem. The need to adopt a family approach was noted:

'The parents are key, above any physios and psychotherapists' (GP.8)

'You find overweight children have overweight parents, so if you tackle the parents, you indirectly tackle the children' (PN. 5)

### The problem of lack of time and pressure of work

GPs identified another important barrier to discussing a child's weight during a consultation as being a limitation of time and resources. They were particularly aware that raising the issue would be time consuming in a busy surgery:

'You only have ten minutes, you just can't do it' (GP.2)

'I don't wish to do it [discuss a child's weight] if I'm busy, cos I know it takes a long time' (GP.11)

'I don't think we should be going out looking for the work. I think that it's more a public health matter, we just don't have the resources to go looking for more work' (GP.6)

## Discussion

### Summary of main findings

The results of this study suggest that the GPs and practice nurses we sampled view childhood obesity as primarily a social or family problem and that the clinician's role is to raise the issue, rather than to manage the problem themselves. The management of childhood obesity was felt to be difficult, primarily due to the sensitive nature of the subject, and the lack of effective interventions. There was a general pessimism that dietary advice would be unsuccessful given the gulf between a healthy diet and what many children ate. We did not detect systematic differences between GPs' views and practice nurses' views.

### Strengths and limitations of the study

One of the strengths of Framework analysis is its flexibility in allowing ideas to be reformulated as the analytical process progresses. However, because it is so "open" it is possible that the researcher's own views, conflicts and prejudices influenced the themes that were subsequently identified from the transcripts. An attempt was made to minimise this by involving in the analysis a second researcher with a different professional background (RA is a non-medical public health specialist whereas OW is a fourth year medical student). Although our results are broadly in keeping with results of similar studies, we can not assume that they are representative of practice nurses and GPs in general. Because our cohort of participants was chosen using opportunistic sampling from only one primary care trust area it may only reflect the views of local clinicians with an interest in obesity research. However, even though this study was of a small scale, it has provided valuable information for the primary care trust and will inform future decision making regarding obesity services.

### Comparison with existing literature

The results of this study appear to be consistent with those found in the majority of the existing literature. There are parallels between the findings of this study and those that relate to adult obesity. GPs and practice nurses felt that ultimately the responsibility for a child's weight fell upon the parents, which is in line with Epstein and Ogden's finding that GPs primarily believed that obesity was the responsibility of the patient [8]. GPs and practice nurses did acknowledge a role in raising the issue of a child's weight and in providing basic diet and exercise advice, again, in line with adult obesity studies [11,12]. A particular emphasis was placed upon the importance of the relationship between the clinician and the family, especially give that childhood obesity is very much viewed as part of a wider family problem. This emphasis on the importance of the doctor-patient relationship is consistent with much published research (e.g. [23,24]), and the concern that raising the issue of a child's weight would threaten this relationship has been noted in a another recent study [18].

Our findings do suggest that, at present, tackling childhood obesity within primary care is extremely challenging to the point that an almost fatalistic perception that "nothing works" has developed. This may reflect the fact that our study was conducted in an area of relatively high socio-economic deprivation, but it is quite possible that these perceptions are held across the UK. The frustration felt by our sample of clinicians may well be shared by many of the consulting parents. Families may feel that they have received dietary and exercise advice many times before, but that this had not solved the problem [25].

### Implications for clinical practice and future research

The Department of Health has recently published a care pathway for the assessment and management of overweight and obese adults, young people and children in primary care [26]. Within this suite of documents guidance is specifically provided to help clinicians in raising the issue of weight with both adults and children, which our study identified as an important barrier to obesity management. However, the real question is "what works in the treatment of childhood obesity?" Clearly there is an urgent need to strengthen the evidence base in this area, and our exploration of the views of GPs and practice nurses in Rotherham suggests that this lack of evidence is an one of the barriers to even raising the issue in the first place. Several studies to determine the effectiveness of psychologically based childhood obesity interventions are currently underway (for example see the [27,28]) and the establishment of a firm evidence base in this area is eagerly awaited. However, if evidence suggests that primary care staff should engage in, for example, more behaviour change or motivational counselling, there is likely to be a significant requirement for increased resources, training and clinician time.

With the forecast rise in childhood obesity, and the Government's stated commitment to address the problem, it is likely that considerable pressure will be placed on GPs and practice nurses to intervene in this arena. The Quality and Outcomes Framework may be one such lever, but given that GPs see childhood obesity as primarily a social or family problem there may be resistance to including more obesity related measures (particularly those related to outcomes) in the contract.

Further research with a larger sample size would be of benefit, and a specific study to determine if the Department of Health's care pathway has assisted primary care clinicians in raising the issue of childhood obesity would be welcome. An analysis of the views of clinicians on the role of targets and incentives in this difficult area would also be of considerable interest.

## Conclusion

The GPs and practice nurses in our sample felt that their role in obesity management was centred upon raising the issue of a child's weight, but that the responsibility of solving the problem lay primarily with the family. Clinicians may find it difficult to make a significant impact on childhood obesity given the sensitivity of the issue, and while the evidence base for effective management remains poor. Implementing additional targets, for example through the QOF, without addressing these fundamental problems may be counterproductive.

## Competing interests

The author(s) declare that they have no competing interests.

## Authors' contributions

JS supervised OW's placement at Rotherham Primary Care Trust, and together with RA and JA they conceived the study. OW conducted the interviews and analysed the data with RA. MS conducted additional analysis and wrote the paper with OW. All authors were responsible for redrafting the final version of the manuscript.

## Pre-publication history

The pre-publication history for this paper can be accessed here:


